# Joint mobilization forces and therapist reliability in subjects with knee osteoarthritis

**DOI:** 10.1179/2042618613Y.0000000033

**Published:** 2013-11

**Authors:** Bradley S Tragord, Norman W Gill, Jason L Silvernail, Deydre S Teyhen, Stephen C Allison

**Affiliations:** 1 Army-Baylor University Doctoral Fellowship in Orthopaedic Manual Physical Therapy, Fort Sam Houston, TX, USA; 2 US Army-Baylor Doctoral Program in Physical Therapy, Fort Sam Houston, TX, USA; 3 First Brigade First Armored Division, Fort Bliss, TX, USA; 4 Telemedicine and Advanced Technology Research Center, US Army Medical Research and Material Command, Ft Detrick, MD, USA

**Keywords:** Manipulation, Musculoskeletal, Physical therapy, Knee joint, Osteoarthritis, Biomechanics

## Abstract

**Objectives:**

This study determined biomechanical force parameters and reliability among clinicians performing knee joint mobilizations.

**Methods:**

Sixteen subjects with knee osteoarthritis and six therapists participated in the study. Forces were recorded using a capacitive-based pressure mat for three techniques at two grades of mobilization, each with two trials of 15 seconds. Dosage (force–time integral), amplitude, and frequency were also calculated. Analysis of variance was used to analyze grade differences, intraclass correlation coefficients determined reliability, and correlations assessed force associations with subject and rater variables.

**Results:**

Grade IV mobilizations produced higher mean forces (*P*<0.001) and higher dosage (*P*<0.001), while grade III produced higher maximum forces (*P* = 0.001). Grade III forces (Newtons) by technique (mean, maximum) were: extension 48, 81; flexion 41, 68; and medial glide 21, 34. Grade IV forces (Newtons) by technique (mean, maximum) were: extension 58, 78; flexion 44, 60; and medial glide 22, 30. Frequency (Hertz) ranged between 0.9–1.1 (grade III) and 1.4–1.6 (grade IV). Intra-clinician reliability was excellent (>0.90). Inter-clinician reliability was moderate for force and dosage, and poor for amplitude and frequency.

**Discussion:**

Force measurements were consistent with previously reported ranges and clinical constructs. Grade III and grade IV mobilizations can be distinguished from each other with differences for force and frequency being small, and dosage and amplitude being large. Intra-clinician reliability was excellent for all biomechanical parameters and inter-clinician reliability for dosage, the main variable of clinical interest, was moderate. This study quantified the applied forces among multiple clinicians, which may help determine optimal dosage and standardize care.

## Introduction

Osteoarthritis (OA) is a disease which commonly affects the knee, often resulting in pain and disability. Lifetime risk of developing symptomatic knee OA is as high as 45%, and although risk factors such as aging, obesity, and female gender are linked to an increased likelihood of developing knee OA, the etiology is not entirely clear.^1^,^2^ Prevalence and costs associated with knee OA have increased substantially over the past decade.^3^,^4^ Identifying and integrating effective interventions into clinical practice are necessary in order to improve functional outcomes and decrease management costs. Commonly recommended conservative intervention strategies include exercise, medications (acetaminophen or NSAIDs), and intra-articular corticosteroid injections.^5^ Unfortunately, acetaminophen and NSAIDs have a small effect size for pain relief and are associated with an increased risk of adverse side effects and increased hospital rates, especially if used in combination.^6^ Single-dose intra-articular corticosteroid injections seem to provide only short-term pain relief, lasting less than 4 weeks. Even when used in multiple doses, corticosteroid injections may not provide long-term improvements in pain relief, physical function, or stiffness.^7^

The importance of general exercise in improving knee joint function and symptoms has been shown in previous studies.^6^,^8^ Zhang *et al.*^6^ reported that using strength training and aerobic exercise as a targeted treatment for knee OA demonstrated a moderate effect size for relief of knee pain. Emerging evidence suggests that using joint mobilization techniques combined with exercise as part of an orthopaedic manual physical therapy approach to treat knee OA can improve pain, stiffness, and function, while decreasing the need for medication and surgery.^9^–^13^

Mobilizations are described as targeted oscillatory manual forces used for many neuromusculoskeletal disorders, including knee OA.^14^ Maitland^15^ described commonly used knee joint mobilization techniques and a grading scheme. Grade I and II mobilizations are performed before joint resistance, while grade III and IV mobilizations are performed into joint resistance. The external application of forces provided by joint mobilizations are thought to be an important part of the mechanism of treatment effect.^15^–^19^ In the lumbar and cervical spine, researchers have analyzed biomechanical parameters such as force, frequency, and amplitude in clinician applied mobilizations.^19^–^26^

Understanding joint mobilization biomechanical parameters and clinician reliability may help refine teaching methods, determine safety of use, establish dose effects, improve mobilization grading schemes, and suggest suitability for use in different patient populations.^20^,^23^,^25^,^27^–^29^ However, there is limited ability to quantify these parameters. Authors have consistently reported poor to fair inter-clinician reliability for joint mobilization biomechanical measurements.^20^,^21^,^23^,^25^,^27^–^29^ Measurement error stems from individual clinicians, instruments and variability of the attribute, notably the patient.^30^ Clinician demographic and anthropometric characteristics may influence mobilization technique application.^26^,^31^ Cook *et al.*^21^ used a force plate to measure spinal mobilization forces and found significant discrepancies between clinicians even when using the same mobilization grade and technique. Hand placement appears to effect mobilization and stiffness assessment;^32^ however, hand or grip strength effects on force application are unknown. In addition to potential clinician influence, instrumentation variation may contribute to the poor to fair reliability trends. The majority of force quantification studies have used indirect methods of measurement, focusing on instrumented tables or devices rather than the force generated at the patient-clinician interface.^21^,^31^,^33^–^36^ Although a few studies have addressed knee stiffness and general biomechanical descriptions of the knee,^37^,^38^ not until recently have results been published addressing specific knee joint mobilization parameters.^39^

Silvernail *et al.*^39^ completed a force quantification study of joint mobilization parameters in patients with knee OA using a pressure platform measurement system. In their study, one experienced manual therapist performed joint mobilization techniques on subjects with knee OA. Intraclass correlation coefficients were above 0.90 for nearly all measured variables suggesting excellent intra-clinician reliability. Force quantification data were provided along with dosage measurement characterized by a force–time integral. Force-time integral is the product of the force over time reported in Newton-seconds and is considered a measure of total mobilization dosage.^39^

Biomechanical parameters must be investigated with multiple clinicians in order to determine generalizability, facilitate communication, identify optimal dosage, and clarify their use as standard of care for patients suffering from knee OA. Given the evidence for manual therapy treatment for knee OA^9^,^12^,^13^ and the newly defined knee force quantification, along with intra-clinician reliability results for a single therapist, we sought to determine the biomechanical parameters and reliability for using similar techniques across multiple clinicians.

This study had three aims. Our first aim was to quantify the biomechanical measures of knee joint mobilizations described in previous clinical trials^12^,^13^ among multiple clinicians for the variables of mean force, maximum force, dosage, peak to peak amplitude, and oscillation frequency in a sample of patients with symptomatic knee OA. In this, we compared grade III and grade IV mobilizations for each technique and hypothesized that grade IV mobilizations would show higher mean forces, similar maximum forces, higher dosage, lower amplitudes, and higher oscillation frequencies than the grade III mobilizations. Our primary variable of clinical interest was dosage. Dosage is an easily understood concept that is frequently addressed in medical literature and may provide valuable understanding in reference to mobilization quantification and prescription. Second, we sought to determine reliability of multiple therapists, including intra- and inter-clinician reliability. Third, we planned to assess clinically relevant correlations among measured biomechanical parameters with our subject and clinician attributes.

## Methods

### Design

This was a descriptive biomechanical study using a cross-sectional observational cohort with prospective sequential enrollment. We quantified biomechanical parameters of knee joint mobilization among six clinicians using a capacitance-based pressure platform (Pliance-x® Novel Electronics Inc., St Paul, MN, USA).

### Setting and participants

Six clinicians were selected through a purposive sample of practicing therapists at Brooke Army Medical Center, San Antonio, Texas. Subjects were recruited from patients referred to physical therapy for symptomatic knee OA at Brooke Army Medical Center from March 2012 to June 2012. All subjects provided written informed consent to participate. The Institutional Review Board at Brooke Army Medical Center approved this study.

### Inclusion and exclusion criteria

Subjects were eligible for military health care and had sufficient English language ability to understand the consent and testing procedures. The treating physical therapist had to judge the subject able to tolerate a knee examination through end range along with repeated bouts of mobilizations targeting 50% (mid-range). Subjects must have met the American College of Rheumatology clinical criteria reporting knee pain for most days of the prior month along with: crepitus with active motion, morning stiffness in the knee ≤ 30 minutes and age ≧38 years; or crepitus and morning stiffness >30 minutes; or no crepitus and bony enlargement of the knee.^40^–^42^ Exclusion criteria included absence of knee joint pain, pain in the knee joint area referred from another region, diagnostic or therapeutic injections to the knee within the last 30 days, history of knee joint replacement on the involved limb, or any standard contraindications to manual therapy (e.g. systemic disease).

### Clinician orientation

Two weeks before data collection, all clinicians attended a 1-hour training session to familiarize themselves with the pressure measurement system. The clinicians did not receive visual feedback from the system as part of their training session because we intended to measure their typical clinical application of manual therapy and avoid any learning effect from using the system.

### Instrumentation

**Figure 1:**
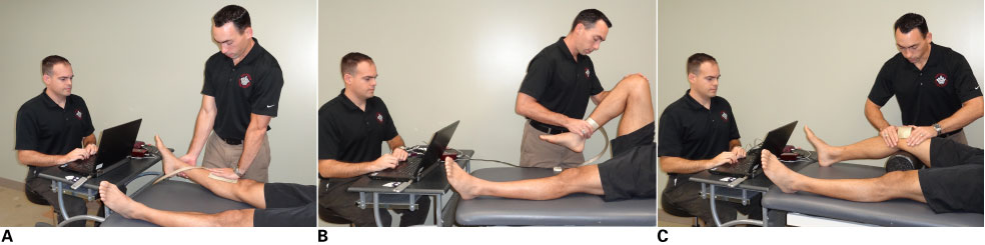
(A) The physiological extension technique. The mobilizing hand is placed on the anterior proximal tibia and the stabilizing hand is place on the posterior calcaneus. The mobilization force is directed posterior creating a physiological extension mobilization; no counterpressure is provided at the heel. (B) The physiological flexion technique. The mobilizing hand is placed on the anterior distal tibia and the stabilizing hand is place on the knee. The mobilization force is directed posterior creating a physiological flexion mobilization; no counterpressure is provided at the knee. (C) The medial patellofemoral accessory glide technique. The distal mobilizing hand is placed on the proximal tibia while the proximal mobilizing hand is placed on the distal femur with thumbs on the lateral aspect of the patella. The mobilization force is directed medial creating an accessory mobilization or glide.

A portable pressure platform was used as a biomechanical data capture package for the measurement of variables (Fig. 1). The system consists of a flexible capacitance-based pressure measuring mat, a multi-channel analyzer, a calibration device, and a software package. The system contains capacitive transducers in a matrix configuration inside the mat. The system is designed with a small, ultra-thin sensing area connected via a conductive strip which does not impede therapist hand placement or feel. The system was calibrated prior to data collection.

### Data collection procedures

Each subject completed intake forms and reported age, height, body weight, and duration of knee symptoms. To describe the effect of their symptoms on function, the subjects completed the Western Ontario and McMaster Universities Osteoarthritis Index (WOMAC) scale.^43^ Subjects also completed the numeric pain rating scale (NPRS) before and immediately after each clinician session to describe the sample and account for any effect pain might have had on the measurements. The 11-point (0–10) NPRS has demonstrated reliability, responsiveness, and validity.^44^,^45^ Subjects rated each clinician on overall level of comfort during each mobilization trial using a 0–10 numeric scale marked ‘very comfortable’ at the left and ‘very uncomfortable’ on the right. This scale is valid, reproducible, and commonly used to evaluate pain as well as comfort or discomfort.^24^,^46^ A radiologist determined the radiographic severity of knee OA in each subject using the 0–4 Kellgren and Lawrence classification system.^47^ The WOMAC, NPRS, Comfort, and Kellgren–Lawrence scales were collected for descriptive purposes only.

A brief manual examination was performed to assess pain and resistance through the range of physiological extension, flexion, and accessory patellar movements, and then to condition the joint for movement. Two clinicians, from a pool of six, were selected based on availability to independently perform the brief examination and apply the joint mobilization techniques and were blinded to the results of the other clinicians. Each clinician determined initial resistance and end range knee extension and applied three different mobilization techniques (physiological tibiofemoral extension, physiological tibiofemoral flexion, medial patellofemoral accessory glide) to the symptomatic knee at mid-range resistance grade III and grade IV in a counterbalanced order. In Maitland’s^15^ expanded grading system, pluses and minuses are used to more precisely define a range of movement with grade III and IV, as used in this trial, targeting 50% of the range of movement between initial and end range resistance. One trial of assessment for knee extension (initial resistance, end range resistance) or mobilization (extension grade IV, extension grade III, flexion grade IV, flexion grade III, medial glide grade IV, medial glide grade III) was followed with a 45-second rest period and a second trial of the same technique at the same grade, resulting in 16 individual measurements for each subject–clinician encounter. Five seconds of data were captured for knee extension assessments and 15 seconds for each mobilization technique×grade combination. Clinicians received no visual or verbal feedback and were blinded to the results of their individual trials. Following data capture, the NPRS and comfort ratings were collected and another clinician immediately repeated the process. Each clinician interaction lasted approximately 30 minutes, for a total of 60 minutes of testing per subject. Seven days later, the subject returned and the process was repeated by two different clinicians from the clinician pool selected based on schedule availability. Overall, there were 1024 measurement trials from the 16 subjects and four clinician sessions per subject.

The independent variables were mobilization grade with two levels (grade III, grade IV) and mobilization technique with three levels (physiological tibiofemoral extension, physiological tibiofemoral flexion, and medial patellofemoral accessory glide). The sensor mat was placed directly on the skin overlying the anterior superior tibia for the extension technique, on the distal tibiofibular area for the flexion technique, and the lateral patella for the medial glide technique. In all cases, the mat was placed between the hand providing the mobilizing force and the subject (Fig. 1).

The dependent variables were mean force, maximum force, dosage, peak-to-peak amplitude, and oscillation frequency. Forces at initial resistance and end range were recorded only for the extension manual assessment. Mean force was the average force measurement across all active sensors on the mat during the trial, and was measured in Newtons. Maximum force was the highest force value from any sensor on the mat during the trial, and was also measured in Newtons. Dosage was characterized by the force-time integral which is the product of the force over time (or area under the curve), recorded in Newton-seconds. Peak-to-peak amplitude was the difference between the highest and lowest force measurement during each trial (measured peak-to-peak), and was reported in Newtons. Oscillation frequency was the number of peaks in the oscillatory pattern of mobilization divided by the 15-second data capture, and was reported in oscillations per second or Hertz.

### Data analysis

An a priori power analysis was performed using G*Power software (Heinrich-Heine University, Dusseldorf, Germany). Based on a study by Silvernail *et al.*,^39^ we used an alpha level of 0.05 and an effect size of 0.7 for the difference in grade III to grade IV dosages. This produced a sample size requirement of 15 patients in order to have 80% power for the study.

Descriptive statistics were calculated for clinician and subject demographics and for the dependent variables of mean force, maximum force, dosage, peak-to-peak amplitude, and oscillation frequency. Kolmogorov–Smirnoff tests were performed on all dependent variables to test the normality assumption.

Mean force, maximum force, dosage, peak-to-peak amplitude, and oscillation frequency were each analyzed using separate 2×3 (grade×technique) repeated-measures analyses of variance with significance level at 0.05 and with *post-hoc* comparisons using the Sidak method. The Greenhouse–Geisser correction was used for any comparisons found to violate the sphericity assumption.

The intraclass correlation coefficient (ICC_3,1_) was calculated for each individual clinician across each dependent variable using the single measurement of trial 1 and trial 2 for initial resistance, end range and every technique–grade combination. The ICC_1,2_ was calculated for the clinician pool for the same dependent variables. Although no clear standards exist, ICC values below 0.75 are generally considered fair to moderate and above 0.75 are considered good reliability.^30^ Response stability of the inter-clinician reliability scores was calculated using the standard error of measurement at the 95% level of confidence.

Correlation statistics were used to evaluate potential associations between maximum forces applied during manual knee extension assessments (initial resistance and end range) and specific variables of interest. A Pearson product–moment correlation was used to assess the relationship between these manual forces with self-reported disability (WOMAC) and pain. Spearman’s rho quantified the relationship between force and the Kellgren–Lawrence score. Additionally, point biserial (for history of hand pain) and Pearson product–moment [age, body mass index (BMI), years of practice, grip and pinch strength] correlations were used to assess the relationships between clinician variables and mean force measurements.

A two-tailed paired *t*-test, with significance level of 0.05, was used to compare the NPRS scores before and after data collection. Data were processed with the Novel Database Medical software (Novel Electronics Corporation) and exported to Microsoft Excel (Microsoft Corporation, Redmond, WA, USA) and SPSS for Windows version 19.0 (SPSS Inc., Chicago, IL, USA) for analysis.

## Results

### Descriptives

The six clinicians in the study consisted of three faculty members and three fellows-in-training working in the Army-Baylor University Doctoral Fellowship in Orthopaedic Manual Physical Therapy, San Antonio, Texas. All clinicians were male with a mean age of 38.7 (10.4) years, height of 1.8 (0.1) m, and weight of 83.2 (7.9) kg. Their clinical experience ranged from 5 to 34 years, with a mean of 12 (11.2) years. Mean grip and pinch strength were 45.8 (4.0) and 9.4 (2.5) kg, respectively. Twenty patients were screened and four were excluded due to recent intra-articular steroid injections, history of systemic disease, or not meeting the American College of Rheumatology knee OA criteria; the remaining 16 patients consented to and completed the study. Characteristics of the study subjects are described in Table 1.

**Table 1:** Subject demographic data

Age (y)	53 (8.9), 41–68
Gender	7 female, 9 male
Height (m)	1.7(0.1), 1.6–1.9
Weight (kg)	88.9 (19.0), 59.0–129.5
BMI (kg/m^2^)	30.1 (4.8), 21.7–41.64
Duration of symptoms (mos)	135.4 (109.1), 26.3–365
WOMAC score (0–240)	96.7 (51.4), 4–175
NPRS (0–10)	3.2 (1.1), 0–6
Kellgren–Lawrence score (0–4)	2 (1.1), 0–3

**Note**: Presented as ‘mean (SD), range’ unless otherwise noted.

Abbreviations: y: years; m: meters; kg: kilograms; BMI: Body Mass Index; mos: months; WOMAC: Western Ontario and McMaster Osteoarthritis Index; NPRS: Numeric Pain Rating Scale.

### Mean force

**Figure 2:**
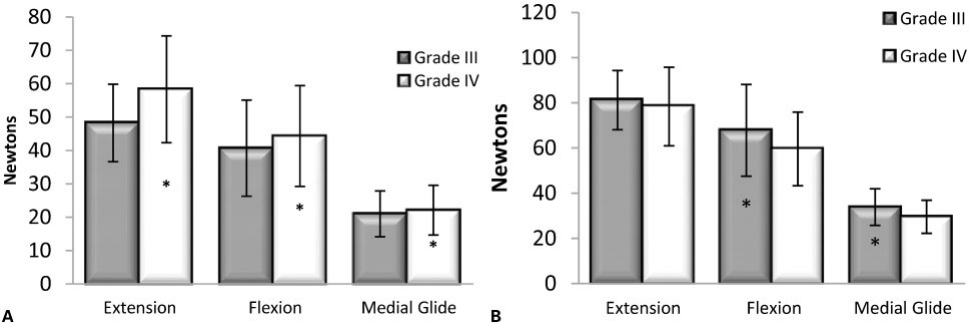
(A) Mean force. Grade IV mean forces were significantly greater than grade III for extension (*P* = 0.001), flexion (*P* = 0.027), and medial glide (*P* = 0.032). (B) Maximum force. Extension grade III and grade IV maximum forces were not significantly different (*P* = 0.352). Maximum force at grade III was greater than grade IV for flexion (*P* = 0.002) and medial glide (*P*<0.001). *Statistically significant difference between grade III and grade IV.

Mean force demonstrated a significant ordinal interaction effect for grade×technique (*F*_2,30_ =  6.95, *P* = 0.003), along with significant main effects for grade (*F*_1,15_ = 25.51, *P*<0.001) and technique (*F*_2,30_ = 51.53, *P*<0.001), as shown in Fig. 2A. Grade IV mean forces were significantly greater than grade III for extension (*P* = 0.001), flexion (*P* = 0.027), and medial glide (*P* = 0.032). Between techniques, the mean force measurements were significantly greater for extension compared to flexion (*P* = 0.046) and medial glide (*P*<0.001), and with flexion greater than medial glide (*P*<0.001). Mean force measurements are reported in Table 2.

**Table 2:** Force and frequency measurements

		Mean force	Max force	Peak-to-peak amplitude	Dosage	Oscillation frequency
Technique	Grade	N	N	N	N s	Hz
Extension	Initial	23.24 (5.00)	23.24 (5.00)	…	…	…
	End range	106.86 (25.78)	106.86 (25.78)	…	…	…
Extension	III	48.26 (11.56)	81.21 (13.13)	60.50 (10.10)	776.43 (185.89)	1.13 (0.13)
	IV	58.34 (16.01)	78.39 (17.41)	37.86 (8.64)	934.19 (259.60)	1.59 (0.11)
Flexion	III	40.70 (14.42)	67.84 (20.34)	46.51 (12.62)	655.14 (237.44)	0.96 (0.16)
	IV	44.34 (15.14)	59.61 (16.25)	27.72 (6.95)	705.71 (262.34)	1.42 (0.12)
Medial-lateral glide	III	21.03 (6.87)	33.89 (8.10)	23.04 (4.51)	339.48 (112.39)	1.12 (0.16)
	IV	22.12 (7.44)	29.59 (7.32)	14.08 (1.84)	351.68 (120.74)	1.58 (0.09)

**Note:** Presented as ‘mean (SD)’ for all measures, N = Newtons, N s =  Newton-seconds, Hz = Hertz.

### Maximum force

Maximum force did not demonstrate a significant interaction effect for grade×technique (*F*_2,30_ = 1.66, *P* = 0.207), but did have a significant main effect for grade (*F*_1,15_ = 16.63, *P* = 0.001) and a significant main effect for technique (*F*_2,30_ = 75.18, *P*<0.001). Between grades, extension grade III and grade IV maximum forces were not significantly different (*P* = 0.352), but there were significant grade differences for flexion (*P* = 0.002) and medial glide (*P*<0.001), as shown in Fig. 2B. With regard to technique, extension was significantly greater than flexion (*P* = 0.016), and medial glide (*P*<0.001), and flexion was significantly greater than medial glide (*P*<0.001). Maximum force measurements are reported in Table 2.

### Dosage

**Figure 3:**
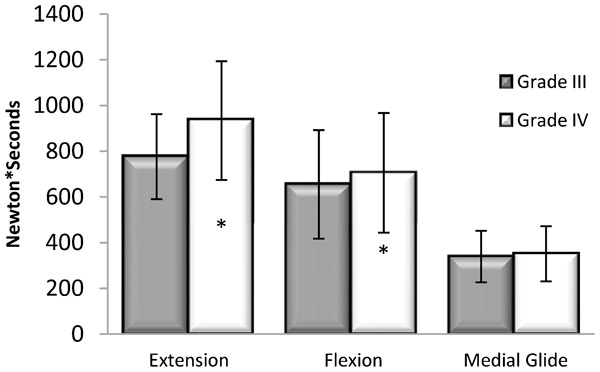
Dosage (force–time integral). Dosage was significantly greater at grade IV for extension (*P* = 0.001) and flexion (*P* = 0.046) compared to grade III, but not statistically different with medial glide (*P* = 0.123). *Statistically significant difference between grade III and grade IV.

Dosage demonstrated a significant ordinal interaction effect for grade×technique (*F*_2,30_ = 8.47, *P* = 0.004), and had significant main effects for grade (*F*_1,15_ = 19.88, *P*<0.001) and technique (*F*_2,30_ = 49.40, *P*<0.001), as shown in Fig. 3. Between grades, dosage was significantly greater at grade IV for extension (*P* = 0.001) and flexion (*P* = 0.046) compared to grade III, but not statistically different with medial glide (*P* = 0.123). Between techniques, the dosage measurements were significantly greater for extension compared to flexion (*P* = 0.047) and medial glide (*P*<0.001) and with flexion greater than medial glide (*P*<0.001). Dosage measurements are reported in Table 2.

### Peak-to-peak amplitude

**Figure 4:**
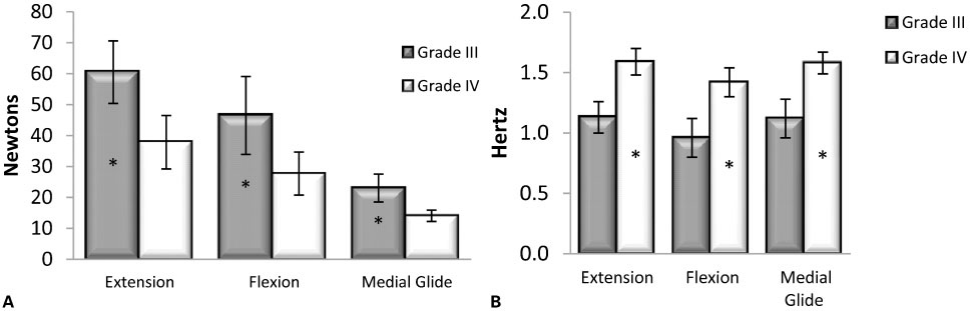
(A) Peak-to-peak amplitude. Peak-to-peak amplitude was consistently greater at grade III for extension, flexion, and medial glide compared to grade IV (*P*<0.001). (B) Frequency. Grade III oscillation frequency measurements were lower than grade IV for all techniques (*P*<0.001). *Statistically significant difference between grade III and grade IV.

Peak-to-peak amplitude demonstrated a significant ordinal interaction effect for grade×technique (*F*_2,30_ = 15.46, *P*<0.001), along with significant main effects for grade (*F*_1,15_ = 135.74, *P*<0.001) and technique (*F*_2,30_ = 80.02, *P*<0.001), as shown in Fig. 4A. Between grades, peak-to-peak amplitude was consistently greater at grade III for extension, flexion, and medial glide compared to grade IV (*P*<0.001). Between techniques, the peak-to-peak amplitude measurements were significantly greater for extension compared to flexion (*P* = 0.002) and medial glide (*P*<0.001) and with flexion greater than medial glide (*P*<0.001). The peak-to-peak amplitudes measurements are reported in Table 2.

### Oscillation frequency

Oscillation frequency did not demonstrate a significant interaction effect for grade×technique (*F*_2,30_ = 0.01, *P* = 0.987), but had significant main effects for grade (*F*_1,15_ = 724.97, *P*<0.001) and technique (*F*_2,30_ = 32.86, *P*<0.001), as shown in Fig. 4B. Grade III oscillation frequency measurements were lower than grade IV for all techniques (*P*<0.001), and the flexion technique showed lower oscillation frequency measurements than extension and medial glide techniques (*P*<0.001). The extension and medial glide were not different (*P* = 0.998). Oscillation frequency measurements are reported in Table 2.

### Reliability

Pooled intra-clinician reliability using ICC_3,1_ for force at initial resistance and end range knee extension, mean force, maximum force, dose, peak-to-peak amplitude, and oscillation frequency ranged between 0.88 and 0.99 (Table 3). For each of the six raters, values of mean intra-clinician reliability across all dependent variables were 0.91, 0.92, 0.94, 0.95, 0.95, and 0.96 (Table 4). Inter-clinician reliability using ICC_1,2_ for initial resistance, end range, mean force, maximum force, dosage, peak-to-peak amplitude, and oscillation frequency ranged between −1.54 and 0.65 (Table 5).

**Table 3:** Mean reliability measures using ICC_3,1_ with 95% confidence intervals

Technique	Grade	Mean force	Maximum force	Peak-to-peak amplitude	Dosage	Oscillation frequency
Extension	Initial	0.93 (0.75–0.98)	0.93 (0.75–0.98)	…	…	…
	End range	0.94 (0.82–0.98)	0.94 (0.82–0.98)	…	…	…
Extension	III	0.88 (0.71–0.96)	0.89 (0.70–0.97)	0.91 (0.73–0.98)	0.89 (0.73–0.97)	0.89 (0.67–0.97)
	IV	0.93 (0.80–0.98)	0.95 (0.84–0.99)	0.92 (0.76–0.97)	0.94 (0.83–0.98)	0.89 (0.63–0.97)
Flexion	III	0.97 (0.90–0.99)	0.95 (0.85–0.99)	0.97 (0.90–0.99)	0.95 (0.82–0.99)	0.90 (0.71–0.97)
	IV	0.96 (0.86–0.99)	0.95 (0.83–0.99)	0.95 (0.84–0.99)	0.97 (0.88–0.99)	0.92 (0.72–0.98)
Medial glide	III	0.95 (0.84–0.99)	0.93 (0.77–0.98)	0.94 (0.80–0.98)	0.95 (0.84–0.99)	0.95 (0.81–0.99)
	IV	0.98 (0.93–0.99)	0.97 (0.89–0.99)	0.98 (0.94–0.99)	0.97 (0.90–0.99)	0.94 (0.75–0.98)

**Note**: Pooled ICC_3,1_ values combining all six clinicians.

**Table 4:** Individual intra-clinician reliability measures using ICC_3,1_ with 95% confidence intervals

	Clinician 1	Clinician 2	Clinician 3	Clinician 4	Clinician 5	Clinician 6
Initial resistance	0.87 (0.63–0.96)	0.96 (0.86–0.99)	0.94 (0.83–0.98)	0.96 (0.83 to 0.99)	0.90 (0.68 to 0.97)	0.93 (0.70 to 0.98)
End range	0.94 (0.82–0.98)	0.95 (0.82–0.99)	0.90 (0.72–0.97)	0.98 (0.92 to 0.99)	0.91 (0.73 to 0.97)	0.98 (0.92 to 0.99)
Mean force	0.94 (0.80–0.98)	0.96 (0.87–0.99)	0.93 (0.81–0.97)	0.99 (0.95 to 0.99)	0.88 (0.71 to 0.96)	0.97 (0.90 to 0.99)
Maximum force	0.92 (0.77–0.98)	0.94 (0.81–0.98)	0.92 (0.78–0.97)	0.97 (0.88 to 0.99)	0.91 (0.76 to 0.97)	0.97 (0.88 to 0.99)
Dose	0.94 (0.82–0.98)	0.97 (0.89–0.99)	0.93 (0.80–0.97)	0.99 (0.94 to 0.99)	0.89 (0.73 to 0.96)	0.95(0.83 to 0.99)
Amplitude	0.94 (0.80–0.98)	0.96 (0.86–0.99)	0.92 (0.80–0.97)	0.98 (0.91 to 0.99)	0.93 (0.78 to 0.98)	0.95 (0.81 to 0.99)
Frequency	0.96 (0.87–0.99)	0.93 (0.77–0.98)	0.93 (0.77–0.98)	0.94 (0.80 to 0.98)	0.95 (0.84 to 0.99)	0.95 (0.81 to 0.99)
**Mean ICC**	0.94 (0.81–0.98)	0.95 (0.84–0.99)	0.92 (0.78–0.97)	0.96 (0.82 to 0.99)	0.91 (0.75 to 0.97)	0.95 (0.83 to 0.99)

**Table 5:** Inter-clinician reliability measures using ICC_1,2_ with 95% confidence intervals

Technique	Grade	Mean force	Maximum force	Peak-to-peak amplitude	Dosage	Oscillation frequency
Extension	Initial	0.48 (−0.10–0.80)	0.48 (−0.10–0.80)	…	…	…
	End range	0.30 (−0.48–0.73)	0.30 (−0.48–0.73)	…	…	…
Extension	III	0.28 (−0.53–0.72)	0.12 (−0.87–0.66)	0.06 (−0.99–0.63)	0.28 (−0.53–0.72)	−0.16 (−1.46–0.55)
	IV	0.53 (0.01–0.82)	0.46 (−0.15–0.79)	0.53 (0.01–0.82)	0.55 (0.05–0.82)	0.29 (−0.51–0.72)
Flexion	III	0.64 (0.23–0.86)	0.67 (0.31–0.87)	0.32 (−0.44–0.73)	0.64 (0.23–0.86)	−0.14 (−1.41–0.56)
	IV	0.53 (0.06–0.83)	0.52 (−0.03–0.81)	−0.04 (−1.2–0.59)	0.65 (0.26–0.86)	−1.54 (−4.38–0.01)
Medial glide	III	0.58 (0.12–0.84)	0.58 (0.10–0.84)	0.18 (−0.75–0.68)	0.60 (0.15–0.84)	−0.20 (−1.55–0.53)
	IV	0.63 (0.20–0.85)	0.56 (0.07–0.83)	−0.57 (−2.33–0.39)	0.65 (0.27–0.87)	−1.54 (−4.39–0.01)

### Correlations

There were no statistically significant relationships between initial resistance and subject BMI (*r* = −0.10, *P* = 0.435), pain (*r* = −0.06, *P* = 0.642), or WOMAC score (*r* = −0.17, *P* = 0.180). Similarly, there were no significant relationships between end range assessment and subject BMI (*r* = −0.14, *P* = 0.276), pain (*r* = −0.09, *P* = 0.476), or WOMAC score (*r* = −0.18, *P* = 0.158). There was no significant relationship between initial resistance and Kellgren–Lawrence score (*r* = −0.23, *P* = 0.074) and a fair, negative, significant relationship between end range and Kellgren–Lawrence score (*r* = −0.028, *P* = 0.023). There were no significant relationships between initial resistance and the clinician variables of BMI (*r* = 0.30, *P* = 0.569), history of pain (*r* = 0.63, *P* = 0.178), grip strength (*r* = 0.45, *P* = 0.374), or pinch strength (*r* = 0.24, *P* = 0.655). There were no significant relationships between end range and the clinician variables of BMI (*r* = 50, *P* = 0.315), history of pain (*r* = −0.16, *P* = 0.758), grip strength (*r* = 0.51, *P* = 0.300), or pinch strength (*r* = 0.57, *P* = 0.237) (Table 6). There were no significant relationships between extension, flexion or medial glide dosage and clinician variables of BMI, history of pain, and grip or pinch strength (Table 7).

**Table 6:** Correlations: clinician and subject variables versus manual assessment

Variable	Initial resistance assessment		End range assessment	
**Subject variables**				
BMI	*r* = −0.10	*P* = 0.435	*r* = −0.14	*P* = 0.276
NPRS	*r* = −0.06	*P* = 0.642	*r* = −0.09	*P* = 0.476
WOMAC	*r* = −0.17	*P* = 0.180	*r* = −0.18	*P* = 0.158
Kellgren–Lawrence	*r* = −0.23	*P* = 0.074	*r* = −0.28*	*P* = 0.023
**Rater variables**				
BMI	*r* = 0.30	*P* = 0.569	*r* = 0.50	*P* = 0.315
Years as PT	*r* = −0.11	*P* = 0.840	*r* = −0.42	*P* = 0.406
Years as OMPT	*r* = 0.001	*P* = 1.00	*r* = −0.55	*P* = 0.260
Hand pain	*r* = 0.63	*P* = 0.178	*r* = −0.16	*P* = 0.758
Grip strength (kg)	*r* = 0.45	*P* = 0.374	*r* = 0.51	*P* = 0.300
Pinch strength (kg)	*r* = 0.24	*P* = 0.655	*r* = 0.57	*P* = 0.237

**Note**: *Statistically significant. Pearson product moment correlations listed for BMI, NPRS, WOMAC, Years as PT, Years as OMPT, Grip, and Pinch strength. Spearman rho correlation for Kellgren–Lawrence. Point–biserial correlation for history of Hand Pain.

Abbreviations: BMI: Body Mass Index; NPRS: Numeric Pain Rating Scale; WOMAC: Western Ontario and McMaster Osteoarthritis Index; kg: kilograms.

**Table 7:** Correlations: clinician variables and dosage (force–time integral)

Rater variables	Extension grade III	Extension grade IV	Flexion grade III	Flexion grade IV	Medial grade III	Medial grade IV
BMI	0.18, *P* = 0.730	0.19, *P* = 0.715	0.24, *P* = 0.642	0.27, *P* = 0.600	0.10, *P* = 0.851	0.18, *P* = 0.732
Years as PT	0.19, *P* = 0.726	0.27, *P* = 0.609	0.47, *P* = 0.346	0.30, *P* = 0.561	0.28, *P* = 0.597	0.15, *P* = 0.778
Years as OMPT	0.07, *P* = 0.890	0.14, *P* = 0.787	0.33, *P* = 0.527	0.16, *P* = 0.763	0.17, *P* = 0.755	0.03, *P* = 0.955
Hand Pain	0.36, *P* = 0.478	0.30, *P* = 0.560	−0.05, *P* = 0.923	−0.17, *P* = 0.748	-0.05, *P* = 0.924	−0.16, *P* = 0.758
Grip strength (kg)	0.56, *P* = 0.250	0.69, *P* = 0.126	0.74, *P* = 0.096	0.73, *P* = 0.099	0.66, *P* = 0.152	0.76, *P* = 0.077
Pinch strength (kg)	0.12, *P* = 0.814	0.09, *P* = 0.870	−0.27, *P* = 0.599	−0.12, *P* = 0.814	−0.05, *P* = 0.929	0.12, *P* = 0.825

**Note**: Pearson product–moment correlations listed for BMI, Years as PT, Years as OMPT, Grip and Pinch strength. Point–biserial correlation for history of Hand Pain.

Abbreviations: BMI: Body Mass Index; kg: kilograms.

### Pain and comfort ratings

Resting knee pain before and after each clinician session was recorded to account for any possible effect of pain on the measures. However, no significant difference between the pre and post NPRS was demonstrated (*t* = 1.31, *P* = 0.248). Mean pain rating (mean±SD) before each clinician session was 2.0±0.33 and after was 2.3±0.18. The mean post-mobilization comfort rating was 1.2±0.31, indicating that the subjects generally viewed the techniques as very comfortable.

## Discussion

The three aims of this study were to quantify biomechanical forces of knee joint manual assessment and mobilizations, determine clinician reliability, and report correlations with clinician and patient attributes. Biomechanical force quantification and observed grade differences were generally consistent with clinical parameters, aiding in the establishment of normative force values for patients with knee OA.^15^,^39^ The peak-to-peak amplitude and oscillation frequency measurements were consistent with previous work on joint mobilization by other authors.^15^,^33^,^39^,^48^ Each of the six clinicians demonstrated excellent intra-clinician reliability (ICC>0.90) for all biomechanical parameters averaged across all techniques and grades. For dosage, our main variable of clinical interest, overall inter-clinician reliability was moderate (ICC = 0.56). However, other variables such as force, frequency, and amplitude demonstrated poor to fair inter-clinician reliability. There were no associations between force measures (initial resistance, end range, or dosage) and clinician attributes (demographics or strength), suggesting that these variables are unlikely to influence precise technique application.

Grade IV mean force measures were greater than grade III across all techniques consistent with the findings of Silvernail *et al.*^39^ However, for maximum force, we found grade III to be greater than grade IV when we would have expected them to be equal. These observed differences (between 1 N or 0.23 pounds and 10 N or 2.27 pounds) were likely too small to be clinically important and suggest that the mobilizations performed in this study were applied at similar points into resistance (Fig. 2).

**Figure 5:**
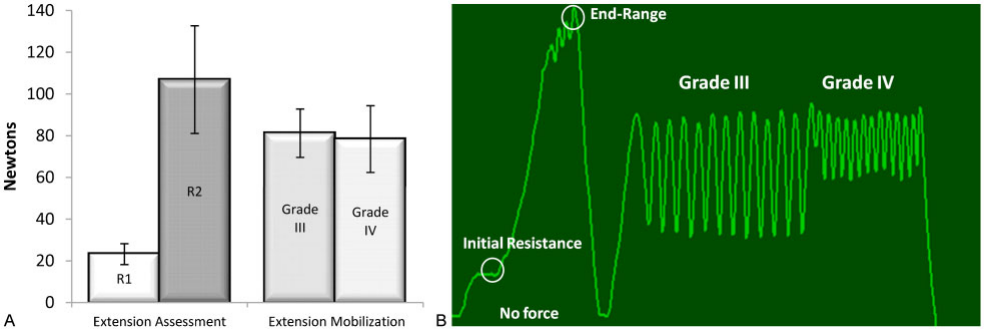
(A) Maximum force quantification of knee extension initial assessment (R1), end range (R2), grade III, and grade IV mobilizations. These maximum forces suggest that grade III and grade IV mobilizations were applied in mid-range. (B) A screen capture of the Pliance system output of force over time. The vertical axis is force (Newtons) and the horizontal axis is time (seconds). This example demonstrates the force at initial resistance, end range, and then a series of grade III followed by grade IV mobilizations at mid-range.

Our biomechanical data provide new insight into the force used to manually examine the knee, particularly knee extension. By first quantifying the full range of knee extension forces (initial resistance to end range), our data demonstrate that the clinicians in this study were able to produce mid-range knee extension mobilizations with reasonable accuracy (Fig. 5), helping to further validate the expanded grading construct described by Maitland.^15^ It is also important to mention that the maximum forces we report are relatively low, supporting their overall safety and feasibility as part of a manual physical therapy approach to knee OA intervention.

We found greater dose delivered with grade IV compared to grade III tibiofemoral mobilizations, similar to Silvernail *et al.*^39^ A higher dosage is expected with a grade IV mobilization since a grade III mobilization will spend more time at lower ranges of resistance due to the large amplitude. Understanding total dosage provides reference for the treating clinician and may help in the application and prescription of therapeutic manual forces. Dosage is an easily understood concept that is frequently addressed in medical literature and clinical practice providing valuable understanding in regard to force quantification, proposed treatment effect and standardization of care. Manual therapists have choices with their interventions and may alter various aspects based on clinical assessment. Although a grade IV mobilization may produce a larger dose for a specific point in resistance over a set period of time, one may find that a grade III technique is better tolerated by the patient. Knee joint mobilizations, as part of a manual physical therapy approach, are low risk and low cost treatments that produce meaningful benefits for people with knee OA.^9^,^12^,^13^ The biomechanical parameters and quantified forces associated among multiple therapists provide important information which may help eventually determine optimal dosage and effectiveness.

Intra-clinician reliability (ICC_3,1_) was excellent across all six clinicians, with mean correlations for each dependent variable ranging from 0.88 to 0.99, consistent with previous results.^26^,^39^,^48^ The average reliability for each individual clinician was also excellent (>0.90). This demonstrates the ability to reproduce mobilizations as an intervention with great precision within the context of the patient–therapist encounter, providing confidence in their ability to provide accurate dosage, progression, and reassessment.

Additionally, we found moderate reliability between clinicians in delivering similar mobilization dosage, though inter-clinician amplitudes and oscillation frequency varied largely. Each of our clinicians was able to self-select amplitude and frequency which may have contributed to the observed variability; however, varying these parameters in the context of a patient–therapist encounter is typical in order to maximize patient comfort, which was high in this study across all clinicians. Our pragmatic study design may have reduced inter-rater reliability but allows for better generalizability. We used symptomatic patients examined on multiple days, and targeted our mobilization forces at the mid-range of resistance (typical for symptomatic patients) which is far more difficult than targeting a well-defined firm end-point. Additionally, we used a ‘clinician pool’ rater scheme consistent with our clinic workflow constraints; this requires the use of a model 1 ICC which typically produces a more conservative estimate of association and agreement.^49^

It was our intent to describe and quantify biomechanical parameters of commonly applied mobilization techniques that replicate actual clinical practice, without artificial standardization. We recognize that higher inter-clinician reliability would be ideal and believe that additional training with the measurement device before testing or combined with real-time biofeedback (verbal and visual) during data collection likely would have improved the results. Although our clinicians completed a short training session prior to the study in order to become competent with the measurement system, formalized feedback training was not completed. Objective and quantitative feedback has been demonstrated to improve consistency and accuracy of joint mobilization performance.^25^,^35^,^50^ Future studies may need to address the optimal time and type of training required to standardize performance across clinicians in symptomatic patient samples.

We collected clinician demographic and manual strength measures to determine if they correlated with force application and found no associations between initial resistance, end range, or dosage with clinician experience, BMI, or strength. This suggests that these clinician attributes are unlikely to influence the application of the examined knee mobilizations. Our findings were consistent with Cook *et al.*,^21^ suggesting that differences are likely intrinsic to the therapist’s application of each mobilization technique (potentially for patient comfort) or some other external variable(s).

Disagreement between clinical, physical, and radiological profiles in knee OA has been reported in the literature and we sought to determine if there was an association between manual assessment forces and subject outcome measures.^51^,^52^ There was poor to no correlation between manual assessment forces with Kellgren–Lawrence, pain, or WOMAC score. One exception was that end range assessment had a fair, but low, negative correlation with Kellgren–Lawrence; however, it is unlikely that this is clinically important.

### Limitations and generalizability

This study sample had a greater proportion of males who were slightly younger than previous trials that demonstrated the effectiveness of knee joint mobilization and exercise.^12^,^13^ The sample used here may not reflect the patient population with symptomatic knee OA who might benefit from orthopaedic manual physical therapy. The measuring instrumentation is another source of potential limitations. The capacitance-based sensors in the mat are designed to measure forces perpendicular to the sensor and may not account for all of the forces applied during the mobilization.

Our study was adequately powered for the biomechanical force quantification, but likely insufficient to yield a robust estimate of reliability or correlation. Therefore, caution must be taken when interpreting those results. However, it is plausible that these results are generalizable to normal clinical practice, as we used symptomatic patients and commonly applied mobilization techniques and grades validated in previous trials.^12^,^13^

## Conclusions

The results of this study help to clarify the mechanical properties of knee joint mobilization and clinician reliability, and evaluate factors that are commonly thought to affect force application. Understanding manual therapy dosage provides valuable insight in regard to force quantification, proposed treatment effect, and effectiveness of care. The high comfort scores indicate that the application of these techniques was well tolerated. Intra-clinician reliability was found to be excellent in this study; and inter-clinician reliability for dosage was found to be moderate. Clinician demographics or strength did not seem to influence application of knee mobilizations. There were poor to no correlations between manual assessment forces with Kellgren–Lawrence, pain, or WOMAC scores. Further exploration and description of joint mobilization techniques using these methods in larger samples will likely provide additional insight to the safe, effective application of joint mobilization in patients with knee OA.

## References

[1] Zhang Y Jordan JM Epidemiology of osteoarthritis. Rheum Dis Clin North Am. 2008;34 (3):515–29 18687270 10.1016/j.rdc.2008.05.007 PMC4384650

[2] Murphy L Schwartz TA Helmick CG Renner JB Tudor G Koch G Lifetime risk of symptomatic knee osteoarthritis. Arthritis Rheum. 2008;59 (9):1207–13 18759314 10.1002/art.24021 PMC4516049

[3] Nguyen US Zhang Y Zhu Y Niu J Zhang B Felson DT Increasing prevalence of knee pain and symptomatic knee osteoarthritis: survey and cohort data. Ann Intern Med. 2011;155 (11):725–32 22147711 10.1059/0003-4819-155-11-201112060-00004 PMC3408027

[4] Lawrence RC Felson DT Helmick CG Arnold LM Choi H Deyo RA Estimates of the prevalence of arthritis and other rheumatic conditions in the United States. Part II. Arthritis Rheum. 2008;58 (1):26–35 10.1002/art.23176 PMC3266664 18163497

[5] American Academy of Orthopaedic Surgeons (AAOS) Treatment of osteoarthritis of the knee (non-arthroplasty). Rosemont, IL: American Academy of Orthopaedic Surgeons (AAOS); 2008

[6] Zhang W Nuki G Moskowitz RW Abramson S Altman RD Arden NK OARSI recommendations for the management of hip and knee osteoarthritis: part III: Changes in evidence following systematic cumulative update of research published through January 2009. Osteoarthr Cartil. 2010;18 (4):476–99 20170770 10.1016/j.joca.2010.01.013

[7] Bellamy N Campbell J Robinson V Gee T Bourne R Wells G Intraarticular corticosteroid for treatment of osteoarthritis of the knee. Cochrane Database Syst Rev. 2006;(2):CD005328 16625636 10.1002/14651858.CD005328.pub2

[8] Fransen M McConnell S Exercise for osteoarthritis of the knee. Cochrane Database Syst Rev. 2008;(4):CD004376 18843657 10.1002/14651858.CD004376.pub2

[9] Jansen MJ Viechtbauer W Lenssen AF Hendriks EJ de Bie RA Strength training alone, exercise therapy alone, and exercise therapy with passive manual mobilisation each reduce pain and disability in people with knee osteoarthritis: a systematic review. J Physiother. 2011;57 (1):11–20 21402325 10.1016/S1836-9553(11)70002-9

[10] Moss P Sluka K Wright A The initial effects of knee joint mobilization on osteoarthritic hyperalgesia. Man Ther. 2007;12 (2):109–18 16777467 10.1016/j.math.2006.02.009

[11] Ottawa panel evidence-based clinical practice guidelines for therapeutic exercises and manual therapy in the management of osteoarthritis. Phys Ther. 2005;85 (9):907–71 16117601

[12] Deyle GD Henderson NE Matekel RL Ryder MG Garber MB Allison SC Effectiveness of manual physical therapy and exercise in osteoarthritis of the knee. A randomized, controlled trial. Ann Intern Med. 2000;132 (3):173–81 10651597 10.7326/0003-4819-132-3-200002010-00002

[13] Deyle GD Allison SC Matekel RL Ryder MG Stang JM Gohdes DD Physical therapy treatment effectiveness for osteoarthritis of the knee: a randomized comparison of supervised clinical exercise and manual therapy procedures versus a home exercise program. Phys Ther. 2005;85 (12):1301–17 16305269

[14] American Physical Therapy Association Guide to physical therapist practice. 2nd ed. Alexandria, VA: APTA; 2001

[15] Maitland GD Hengeveld E Banks K, editors. G. Maitland’s peripheral manipulation. 4th ed. Oxford: Butterworth-Heinemann; 2005

[16] Bialosky JE Bishop MD Price DD Robinson ME George SZ The mechanisms of manual therapy in the treatment of musculoskeletal pain: a comprehensive model. Man Ther. 2009;14 (5):531–8 19027342 10.1016/j.math.2008.09.001 PMC2775050

[17] Bialosky JE George SZ Bishop MD How spinal manipulative therapy works: why ask why? J Orthop Sports Phys Ther. 2008;38 (6):293–5 18515964 10.2519/jospt.2008.0118

[18] Sluka KA Skyba DA Radhakrishnan R Leeper BJ Wright A Joint mobilization reduces hyperalgesia associated with chronic muscle and joint inflammation in rats. J Pain. 2006;7 (8):602–7 16885017 10.1016/j.jpain.2006.02.009

[19] McLean S Naish R Reed L Urry S Vicenzino B A pilot study of the manual force levels required to produce manipulation induced hypoalgesia. Clin Biomech (Bristol, Avon). 2002;17 (4):304–8 10.1016/s0268-0033(02)00017-7 12034124

[20] Binkley J Stratford PW Gill C Interrater reliability of lumbar accessory motion mobility testing. Phys Ther. 1995;75 (9):786–792; discussion 793–5 7659738 10.1093/ptj/75.9.786

[21] Cook C Turney L Ramirez L Miles A Haas S Karakostas T Predictive factors in poor inter-rater reliability among physical therapists. J Manual Manipulative Ther. 2002;10 (4):200–5

[22] de Souza MV Venturini C Teixeira LM Chagas MH de Resende MA Force–displacement relationship during anteroposterior mobilization of the ankle joint. J Manipulative Physiol Ther. 2008;31 (4):285–92 18486749 10.1016/j.jmpt.2008.03.005

[23] Chiradejnant A Maher CG Latimer J Objective manual assessment of lumbar posteroanterior stiffness is now possible. J Manipulative Physiol Ther. 2003;26 (1):34–9 12532136 10.1067/mmt.2003.3

[24] Snodgrass SJ Rivett DA Robertson VJ Manual forces applied during cervical mobilization. J Manipulative Physiol Ther. 2007;30 (1):17–25 17224351 10.1016/j.jmpt.2006.11.008

[25] Snodgrass SJ Rivett DA Robertson VJ Stojanovski E Real-time feedback improves accuracy of manually applied forces during cervical spine mobilisation. Man Ther. 2010;15 (1):19–25 19632877 10.1016/j.math.2009.05.011

[26] Snodgrass SJ Rivett DA Robertson VJ Stojanovski E A comparison of cervical spine mobilization forces applied by experienced and novice physiotherapists. J Orthop Sports Phys Ther. 2010;40 (7):392–401 20592483 10.2519/jospt.2010.3274

[27] Maher C Adams R Reliability of pain and stiffness assessments in clinical manual lumbar spine examination. Phys Ther. 1994;74 (9):801–9; discussion 809–11 8066107 10.1093/ptj/74.9.801

[28] Mootz RD Keating JC Jr Kontz HP Milus TB Jacobs GE Intra- and interobserver reliability of passive motion palpation of the lumbar spine. J Manipulative Physiol Ther. 1989;12 (6):440–5 2628523

[29] Latimer J Lee M Adams RD The effects of high and low loading forces on measured values of lumbar stiffness. J Manipulative Physiol Ther. 1998;21 (3):157–63 9567234

[30] Portney L Watkins M Foundations of clinical research applications to practice. 2nd ed. Upper Saddle River, NJ: Prentice Hall Health; 2000

[31] Chiradejnant A Latimer J Maher CG Forces applied during manual therapy to patients with low back pain. J Manipulative Physiol Ther. 2002;25 (6):362–9 12183694 10.1067/mmt.2002.126131

[32] Maher C Adams R A comparison of pisiform and thumb grips in stiffness assessment. Phys Ther. 1996;76 (1):41–8 8545492 10.1093/ptj/76.1.41

[33] Harms MC Bader DL Variability of forces applied by experienced therapists during spinal mobilization. Clin Biomech (Bristol, Avon). 1997;12 (6):393–9 10.1016/s0268-0033(97)00023-5 11415748

[34] Harms MC Innes SM Bader DL Forces measured during spinal manipulative procedures in two age groups. Rheumatology (Oxford). 1999;38 (3):267–74 10325666 10.1093/rheumatology/38.3.267

[35] Lee M Moseley A Refshauge K Effect of feedback on learning a vertebral joint mobilization skill. Phys Ther. 1990;70 (2):97–102; discussion 103–4 2296617 10.1093/ptj/70.2.97

[36] Threlkeld AJ The effects of manual therapy on connective tissue. Phys Ther. 1992;72 (12):893–902 1454865 10.1093/ptj/72.12.893

[37] Burks K Keegan K Objective measurement of stiffness in knee osteoarthritis. Orthop Nurs. 2006;25 (4):244–50 16900068 10.1097/00006416-200607000-00005

[38] Hamstra-Wright KL Swanik CB Ennis TY Swanik KA Joint stiffness and pain in individuals with patellofemoral syndrome. J Orthop Sports Phys Ther. 2005;35 (8):495–501 16187510 10.2519/jospt.2005.35.8.495

[39] Silvernail JL Gill NW Teyhen DS Allison SC Biomechanical measures of knee joint mobilization. J Manual Manipulative Ther. 2011;19 (3):162–71 10.1179/2042618611Y.0000000012 PMC3143014 22851879

[40] Wu CW Morrell MR Heinze E Concoff AL Wollaston SJ Arnold EL Validation of American College of Rheumatology classification criteria for knee osteoarthritis using arthroscopically defined cartilage damage scores. Semin Arthritis Rheum. 2005;35 (3):197–201 16325660 10.1016/j.semarthrit.2005.06.002

[41] Altman R Asch E Bloch D Bole G Borenstein D Brandt K Development of criteria for the classification and reporting of osteoarthritis. Classification of osteoarthritis of the knee. Diagnostic and Therapeutic Criteria Committee of the American Rheumatism Association. Arthritis Rheum. 1986;29 (8):1039–49 3741515 10.1002/art.1780290816

[42] Altman RD Criteria for classification of clinical osteoarthritis. J Rheumatol Suppl. 1991;27:10–2 2027107

[43] Bellamy N Buchanan WW Goldsmith CH Campbell J Stitt LW Validation study of WOMAC: a health status instrument for measuring clinically important patient relevant outcomes to antirheumatic drug therapy in patients with osteoarthritis of the hip or knee. J Rheumatol. 1988;15 (12):1833–40 3068365

[44] Jensen MP Turner JA Romano JM What is the maximum number of levels needed in pain intensity measurement? Pain. 1994;58 (3):387–92 7838588 10.1016/0304-3959(94)90133-3

[45] Jensen MP Turner JA Romano JM Fisher LD Comparative reliability and validity of chronic pain intensity measures. Pain. 1999;83 (2):157–62 10534586 10.1016/s0304-3959(99)00101-3

[46] de Looze MP Kuijt-Evers LF van Dieën J Sitting comfort and discomfort and the relationships with objective measures. Ergonomics. 2003;46 (10):985–97 12850935 10.1080/0014013031000121977

[47] Kellgren JH Lawrence JS Radiological assessment of osteo-arthrosis. Ann Rheum Dis. 1957;16 (4):494–502 13498604 10.1136/ard.16.4.494 PMC1006995

[48] Snodgrass SJ Rivett DA Robertson VJ Stojanovski E Forces applied to the cervical spine during posteroanterior mobilization. J Manipulative Physiol Ther. 2009;32 (1):72–83 19121465 10.1016/j.jmpt.2008.09.012

[49] Shrout PE Fleiss JL Intraclass correlations: uses in assessing rater reliability. Psychol Bull. 1979;86 (2):420–8 18839484 10.1037//0033-2909.86.2.420

[50] Latimer J Lee M Adams R The effect of training with feedback on physiotherapy students’ ability to judge lumbar stiffness. Man Ther. 1996;1 (5):266–70 11440516 10.1054/math.1996.0277

[51] Schiphof D de Klerk BM Koes BW Bierma-Zeinstra S Good reliability, questionable validity of 25 different classification criteria of knee osteoarthritis: a systematic appraisal. J Clin Epidemiol. 2008;61 (12):1205–15 18782658 10.1016/j.jclinepi.2008.04.003

[52] Wolfe F Lane NE The longterm outcome of osteoarthritis: rates and predictors of joint space narrowing in symptomatic patients with knee osteoarthritis. J Rheumatol. 2002;29 (1):139–46 11824950

